# Toxicologic Evaluation for Amorphous Silica Nanoparticles: Genotoxic and Non-Genotoxic Tumor-Promoting Potential

**DOI:** 10.3390/pharmaceutics12090826

**Published:** 2020-08-29

**Authors:** Gwang-Hoon Lee, Yun-Soon Kim, Euna Kwon, Jun-Won Yun, Byeong-Cheol Kang

**Affiliations:** 1 Graduate School of Translational Medicine, Seoul National University College of Medicine, Seoul 03080, Korea; ghl@dgmif.re.kr; 2 Laboratory Animal Center, Daegu-Gyeongbuk Medical Innovation Foundation, Daegu 41061, Korea; 3 Department of Experimental Animal Research, Biomedical Research Institute, Seoul National University Hospital, Seoul 03080, Korea; gys0804@nate.com (Y.-S.K.); eunakwon@snuh.org (E.K.); 4 Department of Biotechnology, The Catholic University of Korea, Bucheon 14662, Korea; 5 Designed Animal Resource Center, Seoul National University, Pyeongchang-gun 25354, Korea

**Keywords:** nanoparticles, amorphous silica, genotoxicity, gap junctional intercellular communication, tumor promoting potential

## Abstract

Amorphous silica nanoparticles (SiO_2_NPs) have been widely used in medicine including targeted drug/DNA delivery, cancer therapy, and enzyme immobilization. Nevertheless, SiO_2_NPs should be used with caution due to safety concerns associated with unique physical and chemical characteristics. The objective of this study was to determine the effects of SiO_2_NPs on genotoxic and non-genotoxic mechanisms associated with abnormal gap junctional intercellular communication (GJIC) in multistage carcinogenesis. The SiO_2_NPs exhibited negative responses in standard genotoxicity tests including the Ames test, chromosome aberration assay, and micronucleus assay. In contrast, the SiO_2_NPs significantly induced DNA breakage in comet assay. Meanwhile, SiO_2_NPs inhibited GJIC based on the results of scrape/loading dye transfer assay for the identification of non-genotoxic tumor-promoting potential. The reduction in expression and plasma membrane localization of Cx43 was detected following SiO_2_NP treatment. Particularly, SiO_2_NP treatment increased Cx43 phosphorylation state, which was significantly attenuated by inhibitors of extracellular signal-regulated kinases 1/2 (ERK1/2) and threonine and tyrosine kinase (MEK), but not by protein kinase C (PKC) inhibitor. Taken together, in addition to a significant increase in DNA breakage, SiO_2_NP treatment resulted in GJIC dysregulation involved in Cx43 phosphorylation through the activation of mitogen-activated protein kinase (MAPK) signaling. Overall findings of the genotoxic and non-genotoxic carcinogenic potential of SiO_2_NPs provide useful toxicological information for clinical application at an appropriate dose.

## 1. Introduction

Nanoparticles are materials measuring less than 100 nm in at least one dimension. Due to the unique physical and chemical properties of nanomaterials compared with their micro-sized counterparts, nanotechnology is one of the most rapidly growing technologies in various fields including food, agriculture, energy, materials, healthcare, and medicine [1,2,3]. Nonetheless, nanoparticles are associated with major safety concerns following broader human exposure. Since the ultra-small size of nanomaterial facilitates its diffusion across biological barriers [4], smaller particles with a larger surface area are more toxic with a higher absorption rate than the larger ones [5,6]. Indeed, Warheit et al. [7] found that nanoscale TiO_2_ induces transient inflammatory cells after pulmonary instillation in rats. Kim et al. [8] found that silver nanoparticles trigger oxidative stress in human hepatoma cells. Raghunathan et al. [9] reported chrome nanoparticle-induced genotoxicity by inducing reactive oxygen species. Exposure to zinc oxide nanoparticles led to cytotoxicity, genotoxicity, and inflammatory responses in human nasal mucosa cells [10]. Meanwhile, Magaye et al. [11] reported genotoxicity and carcinogenicity of cobalt, nickel, and copper-based nanoparticles.

Silicon dioxide, also known as silica, exists in three forms: crystalline, amorphous, and fused silica. Among them, there is extensive evidence that crystalline silica is toxic. Excessive exposure to crystalline silica has been known to increase the risk of pulmonary diseases such as silicosis and lung cancer [12]. Fused silica particles are reported to induce interleukin-1β (IL-1β) release from lipopolysaccharide (LPS)-primed mouse macrophage-like cell line RAW264.7 [13]. As another form of silica, amorphous silica has a wide range of applications including food and cosmetic industries as well as in medicine via targeted drug/DNA delivery, cancer therapy, enzyme immobilization, and in dentistry as an abrasive agent [14,15,16,17,18]. Although amorphous silica has been considered to be a less toxic form, its toxicity is still highly disputed. Ryu et al. [19] did not find any toxicity or change in organs following exposure to amorphous silica nanoparticles (SiO_2_NPs). Uboldi et al. [15] also have shown that SiO_2_NPs did not induce cytotoxicity, cell transformation, or genotoxicity in mouse fibroblast cells. In contrast, SiO_2_NPs are known to cause cytotoxicity, resulting in oxidative stress and apoptosis of epithelial cells in human skin and lung [16]. Guichard et al. [20] demonstrated cytotoxicity and genotoxicity of SiO_2_NPs in vitro.

**Figure 1:**
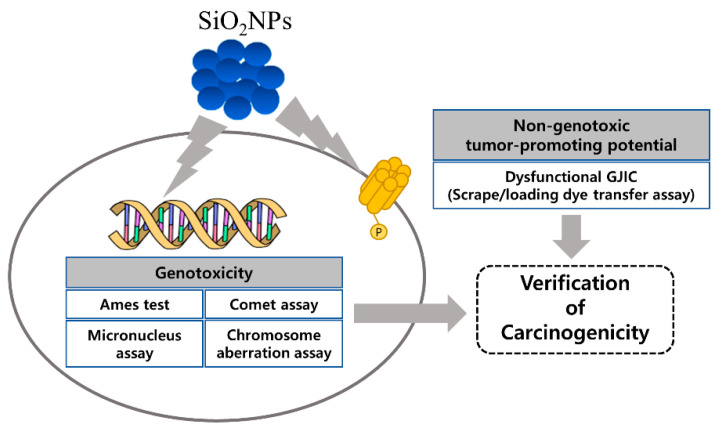
Schematic representation of the experimental design.

Based on the current knowledge on the genotoxic and carcinogenic potential of various nanoparticles [11], the present study was designed to provide conclusive information on the genotoxic potential of SiO_2_NPs using a battery of four standard genotoxicity tests including the Ames test, in vitro chromosome aberration test, in vivo micronucleus test, and additional in vitro comet assay, in compliance with the Good Laboratory Practices for toxicity test guidance issued by the Ministry of Food and Drug Safety [21]. Further, to investigate the non-genotoxic carcinogenic potential of SiO_2_NPs, we also conducted gap junctional intercellular communication (GJIC) analysis, a rapid and simple protocol to detect tumor promoters [22,23,24,25]. Gap junction consists of two intercellular hemichannels called connexons. A connexon is composed of six connexins that directly link the cytoplasm of neighboring cells and facilitate the passage of ions and signaling molecules, nucleotides, inositol triphosphate, Ca^2+^, second messengers, and other essential cellular components to maintain homeostasis, cell growth, proliferation, differentiation and other physiological events [26]. Thus, GJIC dysfunction can cause loss of homeostasis, resulting in carcinogenesis [24,25]. Using this approach, we identified the tumor-promoting potential of the SiO_2_NPs and its mechanism associated with abnormal GJIC. Based on the genotoxic and non-genotoxic carcinogenic potential of the SiO_2_NPs, we provide comprehensive preclinical data for the clinical use (Figure 1).

## 2. Materials and Methods

### 2.1. Physicochemical Characterization

Particle size and morphology of SiO_2_NPs (Nanostructured and Amorphous Materials Inc., Houston, TX, USA) were evaluated with a transmission electron microscope (TEM) (JEOL 2010F, JEOL, Tokyo, Japan). Stock suspensions of particles were prepared in ddH_2_O by sonication for 1 h (pulse on 30 s/pulse off 30 s each cycle) in a dark room to prevent the effect of light. This suspension was pipetted onto the formvar/carbon-coated TEM grid. After droplets were left to dry at room temperature, they were photographed. The sizes of 20 particles on the grid were measured and their average value was calculated. Scanning electron microscopy (SEM) was utilized to study the morphology of SiO_2_NPs by using an SU8010 High Resolution Field Emission Scanning Electron Microscope (Hitachi, Ltd., Tokyo, Japan). Elemental composition of SiO_2_NPs was confirmed and quantified using the energy dispersive X-ray detection (EDX) feature of the SEM. The hydrodynamic size and zeta potential of SiO_2_NPs were determined by dynamic light scattering (DLS) with a Zetasizer Nano-ZS instrument (Malvern, Herremberg, Germany). The DLS measurement was performed 30 min after suspension preparation at a concentration of 1 mg/mL.

### 2.2. Ames Test

Bacterial reverse mutation assay (Ames test) was conducted in accordance with the Organization for Economic Cooperation and Development (OECD) guideline 471 [27] using characterized histidine-requiring strains of *Salmonella typhimurium* (TA98, TA100, TA1535, TA1537; Ministry of Food and Drug Safety, Osong, Korea), and *Escherichia coli* (WP2uvrA; MOLTOX, Boone, NC, USA) to investigate the potential mutagenicity of SiO_2_NPs. These test strains were treated with SiO_2_NPs at doses of 1250, 2500, and 5000 μg/100 μL/plate with or without an exogenous metabolic activation (S9 mix) in the dark at 37 °C for 48 h. The following compounds were used as positive controls: 2-nitrofluorene, sodium azide, mitomycin C, 9-aminoacridine, and 2-aminoanthracene (Sigma-Aldrich, St. Louis, MO, USA). A ≥2-fold increase and a concentration-dependent increase in the number of revertant colonies relative to the negative control were considered positive for mutagenicity.

### 2.3. In Vitro Chromosomal Aberration Study

Chromosomal aberrations were analyzed using Chinese hamster lung (CHL) fibroblasts, a widely used test system for in vitro genotoxicity studies, according to the OECD guideline 473 [28]. CHL fibroblasts were treated with SiO_2_NPs (133.38, 153.39, 176.4 µg/mL), mitomycin C (0.1 µg/mL), and cyclophosphamide (5 µg/mL) in the presence or absence of S9 mix for 6 h or 24 h in a 5% CO_2_ atmosphere at 37 °C. The cells were washed and incubated in complete medium for an additional 18 h. After the addition of colcemid (Gibco, Carlsbad, CA, USA) at a final concentration of 0.2 μg/mL for 2 h, the cells were swollen in a hypotonic solution, fixed in 3:1 methanol/glacial acetic acid, and stained with 4% Giemsa.

### 2.4. In Vivo Bone Marrow Micronucleus Test

An in vivo bone marrow micronucleus test was conducted using male ICR(CrljOri:CD1) mice (Orient Bio, Seongnam, Korea) aged 7 weeks according to the OECD guideline 474 [29]. The animal experiments were reviewed and approved by the Institutional Animal Care and Use Committee of the Biomedical Research Institute at the Seoul National University Hospital (Identification code: 12-0373, Approved date: 1 January 2013. SiO_2_NPs were administered daily via oral gavage for 4 days at doses of 500, 1000, and 2000 mg/kg of body weight. Mitomycin C was administered intraperitoneally at a dose of 2 mg/kg of body weight as a positive control. All mice were sacrificed at 24 h after treatment, and femurs were removed to obtain bone marrow cells. The cells were centrifuged, smeared onto slides, dried, and fixed in methanol. The fixed slides were stained with 5% Giemsa. A total of 2000 polychromatic erythrocytes (PCEs) were counted to determine the frequency of micronucleated polychromatic erythrocytes (MNPCEs). Additionally, the ratio of PCEs to PCEs + normochromatic erythrocytes (NCEs), where the NCEs denote the normochromatic erythrocytes, was calculated by counting a total of 1000 erythrocytes to determine the possibility of bone marrow cytotoxicity [30].

### 2.5. In Vitro Comet Assay

The comet assay was performed with CHL cells to evaluate the DNA damage induced by SiO_2_NPs as described by Singh et al. [31] and Olive et al. [32]. The cells were exposed to SiO_2_NPs at 37.5, 75, and 150 μg/mL for 4 or 24 h in the presence or absence of S9 mix. Ethyl methanesulfonate (EMS) at 500 µg/mL was used as a positive control. The treated cells were collected, mixed with a low melting point agarose (0.8%), spread onto pre-coated glass slides, covered with a glass coverslip, and placed at 4 °C. After the coverslip was removed, the slides were placed in a lysis solution (2.5 M NaCl, 100 mM EDTA, 10 mM Tris-HCl, 1% Triton X-100, 10% (*v*/*v*) DMSO) and unwinding buffer (1 mM EDTA, 300 mM NaOH, pH > 13), and subjected to electrophoresis. Next, the slides were neutralized with 0.4 M Tris-HCl (pH 7.5) for 10 min, stained with ethidium bromide (2 µg/mL), and analyzed using fluorescence microscopy. DNA damage was determined by % tail DNA and Olive tail moment (% tail DNA × tail movement length) based on the random scoring of 100 nuclei per slide.

### 2.6. Cell Culture and Treatments for GJIC Analysis

Cytotoxicity was measured using a trypan blue exclusion assay as described by Strober [33] with some modifications. In brief, WB-F344 rat liver epithelial stem-like cells were seeded onto a 24-well plate and grown in D-medium (Gibco) supplemented with 5% fetal bovine serum (Gibco), 0.5% penicillin-streptomycin-neomycin antibiotic mixture (Gibco), sodium bicarbonate (Amresco, Solon, OH, USA), sodium pyruvate (Sigma-Aldrich), d-glucose (Sigma-Aldrich), and sodium chloride (Sigma-Aldrich) at 37 °C in a 5% CO_2_ humidified incubator. To determine the concentration that results in 70–80% viability compared with the negative control, WB-F344 cells were exposed to SiO_2_NPs at different concentrations up to 5000 µg/mL for 24 h. After exposure to a mixture of 0.4% trypan blue, viability was determined as the percentage of cells with clear cytoplasm (viable cells) versus cells that contained trypan blue in the cytoplasm (dead cells). For the time-course experiments investigating the function of GJIC using scrape loading/dye transfer (SL/DT) assay, WB-F344 cells were treated with SiO_2_NPs at a dose of 5000 μg/mL for 3, 6, 12, and 24 h. In the dose-dependent study, the cells were treated with 200, 1000, and 5000 µg/mL of SiO_2_NPs for 12 h. As a positive control, 12-O-tetradecanocylphorbol-13-acetate (TPA) was added at a dose of 10 ng/mL. To identify the mechanism of SiO_2_NP-induced GJIC inhibition, WB-F344 cells were pretreated with 50 µM extracellular signal-regulated kinase (ERK) inhibitor PD98059 (Sigma-Aldrich), 10 µM mitogen-activated protein kinase kinase (MEK) inhibitor U0126 (Sigma-Aldrich), and 10 µM protein kinase C (PKC) inhibitor bisindolylmaleimide I (BIM I) (Calbiochem, San Diego, CA, USA) for 30 min before exposure to 5000 µg/mL SiO_2_NPs for 12 h.

### 2.7. SL/DT Assay for GJIC Analysis

The SL/DT assay was conducted to measure GJIC using Lucifer yellow dye migration through all connexin channels with the scrape and load technique [34]. After cells were washed 3 times with Dulbecco’s phosphate-buffered saline (D-PBS) without Ca^2+^ and Mg^2+^, 0.05% Lucifer yellow dye (Sigma-Aldrich) dissolved in D-PBS was added to cells and six scrapes were made with a surgical steel blade. After 9 min of incubation at room temperature in the dark, the Lucifer yellow was discarded. Cells were washed 3 times with D-PBS and fixed with 4% formaldehyde solution. The GJIC activity was analyzed by monitoring the extent of diffusion of the fluorescent dye Lucifer yellow from the scrape line into adjacent cells through functional gap junctions using an inverted fluorescent microscope (IX61, Olympus, Tokyo, Japan).

### 2.8. Immunofluorescence Staining of Cx43

Immunofluorescence staining was carried out to verify the quantification and localization of Cx43 protein. WB-F344 cells seeded on the 8-well chamber slide were blocked with 3% bovine serum albumin (BSA) (Amresco) in PBS containing 0.05% Tween 20 for 1 h at room temperature. After the supernatant was discarded, cells were incubated overnight with rabbit polyclonal anti-Cx43 antibody (Sigma-Aldrich) at 4 °C. After washing 3 times, the cells were incubated with goat anti-rabbit immunoglobulin G (IgG) secondary antibody (Molecular Probes, Eugene, OR, USA) for 1 h at room temperature. Cells were washed 3 times with D-PBS and fixed with 4% paraformaldehyde for 20 min at room temperature. Samples were mounted and photographed with an inverted fluorescent microscope.

### 2.9. Western Blot Analysis

Western blot was used to measure the phosphorylation of Cx43, ERK, MEK, and PKC. Proteins were extracted with radioimmunoprecipitation (RIPA) buffer (Millipore, Bedford, MA, USA) containing 0.1% protease inhibitor (Sigma-Aldrich) and phosphatase inhibitor (Sigma-Aldrich), and centrifuged at 13,000 rpm for 20 min at 4 °C. The supernatants were collected, and the protein contents were determined using the bicinchoninic acid (BCA) protein assay kit (Pierce Biotechnology, Rockford, IL, USA), followed by 12% SDS-PAGE of 20 µg proteins and transfer to polyvinylidene difluoride membranes (Bio-Rad, Richmond, CA, USA). The membrane was incubated with anti-Cx43 (Sigma-Aldrich), anti-pERK1/2 (Cell signaling Technology, Beverly, MA, USA), anti-ERK1/2 (Cell signaling Technology), anti-pMEK (Cell signaling Technology), anti-MEK (Cell signaling Technology), anti-PKC (Santa Cruz Biotechnology, Santa Cruz, CA, USA), or anti-pPKC (Biovision Inc., Milpitas, CA, USA) antibodies overnight at 4 °C. Signals were detected with a chemiluminescence kit (GE Healthcare, Buckinghamshire, UK) according to the manufacturer’s instructions. The relative band intensity and phosphorylation status were quantified with an image-analysis program using Image J (NIH, Bethesda, MD, USA) or Scion Image (NIH).

### 2.10. Statistical Analysis

The data were expressed as means ± SD. Statistical analysis was performed using a one-way ANOVA or Student’s *t*-test using SPSS software version 24 (SPSS Inc., Chicago, IL, USA). *p* values ≤ 0.05 were considered statistically significant.

## 3. Results

### 3.1. Characterization of SiO_2_NPs

**Figure 2:**
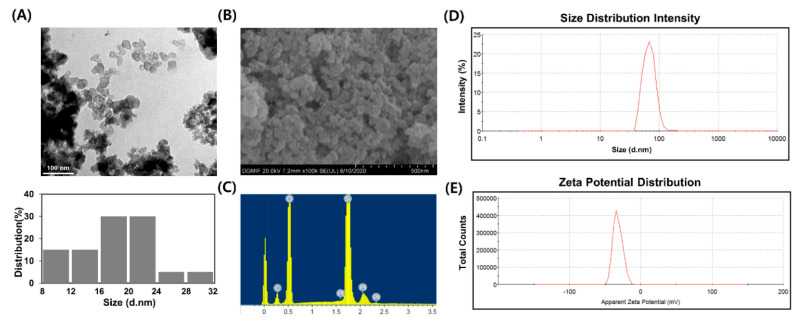
Characterization of amorphous silica nanoparticles (SiO_2_NPs). (**A**) Representative TEM image and size distribution histogram of SiO_2_NPs. (**B**) Representative SEM image of the SiO_2_NPs. (**C**) energy dispersive X-ray detection (EDX) analysis of SiO_2_NPs. (**D**,**E**) Representative dynamic light scattering (DLS) measurement of SiO_2_NPs for (**D**) hydrodynamic size and (**E**) zeta potential.

The characterization of nanoparticles is a basic step in the evaluation of potential toxicity. As a nanoparticle is characterized by less than 100 nm at least in one dimension, the size and morphology of SiO_2_NPs were verified by TEM. The morphology of the SiO_2_NPs used in this study was spherical with no impurity (Figure 2A). 

The measured TEM-size of SiO_2_NPs was 18.54 ± 5.05 nm (Table 1). Scanning electron microscopy analysis provided further insight into morphology of SiO_2_NPs and the SiO_2_NPs were spherical in shape (Figure 2B). For the confirmation of elemental analysis, SiO_2_NPs was subjected to the EDX analysis. The results indicated that SiO_2_NPs were composed of the highest purity because no peak belonging to impurity was detected (Figure 2C). Platinum and carbon were detected because SiO_2_NPs was coated with platinum before EDX analysis and SiO_2_NPs was pasted on carbon tape during detecting, respectively. DLS can also be used to evaluate the hydrodynamic size and zeta potential [10]. Kaszuba and Connah [35] indicated that the concentration of particles can affect light scattering when measuring hydrodynamic size by DLS. If the concentration of the particle is too high, multiple scattering effect can occur, which leads to reduction in hydrodynamic size. Therefore, in this study, DLS analysis was performed using suspensions of SiO_2_NPs at a concentration of 1 mg/mL, not 50 mg/mL used as the highest concentration in the Ames test. Our finding showed a hydrodynamic size of 69.35 nm (Figure 2D and Table 1), indicating that SiO_2_NPs may form minimal agglomeration/aggregation and were appropriate for nanotoxicity studies. The zeta potential of SiO_2_NPs in water diluted to 0.1 mg/mL was −32.2 mV (Figure 2E and Table 1). The absolute zeta potential of 30 mV or more is stable enough to overcome aggregation caused by the action of Van der Waals forces [36].

### 3.2. SiO_2_NPs Exposure Results in Ames Test

The results of the Ames test with *S. typhimurium* TA98, TA100, TA1535, TA1537, and *E. coli* WP2uvrA, which is used to determine the mutagenicity potential of SiO_2_NPs, are presented in Table 2. As expected, standard mutagens used as positive controls (2-nitrofluorene, sodium azide, mitomycin C, 9-aminoacridine, and 2-aminoanthracene) for the respective strains caused a significant increase in the number of His^+^revertant colonies in the presence or absence of the S9 mix, indicating that the test conditions and the metabolic activation system were adequate. In contrast, no significant increases in the number of revertants were observed in all bacterial strains with or without S9 mix upon treatment with SiO_2_NPs at doses of 1250, 2500, and 5000 μg/100 μL/plate selected based upon preliminary results from cytotoxicity testing (Supplementary Materials Table S1).

### 3.3. SiO_2_NPs Exposure Results In Vitro Chromosomal Aberration Assay

As shown in a preliminary 3-[4,5-dimethylthiazol-2-yl]-2,5 diphenyl tetrazolium bromide (MTT) assay in CHL cells (Table S2), dose-dependent cytotoxicity was observed after SiO_2_NP treatments. Although it is possible that bioavailabilities higher than 100% in the treatments of low doses of SiO2NPs might be due to magnetic properties and catalytic activity [37], redox properties [38], or lixiviation of nanoparticle ions [39], these were not very high (107.48–114.37%). Based on the results of the MTT assay, 176.4 μg/mL of SiO_2_NPs with a cell survival rate of 73.21% was selected as the highest concentration for an in vitro chromosomal aberration assay. The positive controls, 0.1 µg/mL mitomycin C (−S9) and 5 µg/mL cyclophosphamide (+S9), exhibited a significant increase in the incidence of chromosomal aberrations, including breaks, fragments, and exchanges, compared with the negative control group. Under this valid condition, SiO_2_NPs did not increase the incidence of chromosomal aberrations with or without exogenous metabolic activation at doses of 133.38, 153.39, and 176.4 µg/mL (Table 3).

### 3.4. SiO_2_NPs Exposure Results In Vivo Bone Marrow Micronucleus Assay

In vivo micronucleus assay was conducted to detect the structural (clastogenic) and numerical (aneugenic) chromosome changes following SiO_2_NP treatments. Although the nanoparticles can be introduced into the biological systems through different routes (oral, inhalation, intravenous, etc.), the oral route was chosen as the most likely route of exposure to SiO_2_NPs in this in vivo genotoxicity assay since SiO_2_NPs have been recently reported as an oral drug delivery system [18]. Throughout the study period, no mortality, clinical signs, or abnormalities in body weight (Table S3) were observed following exposure to SiO_2_NPs up to 2000 mg/kg based on the maximum limit dose recommended in OECD. The mean ratios of the cytotoxicity index PCEs/(PCEs + NCEs) were above 39.8% in all the tested groups including negative and positive controls (Table 4), indicating a lack of cytotoxicity associated with SiO_2_NP treatment under the current test condition [30]. While a statistically significant increase in the number of MNPCEs was detected in the group treated with mitomycin C relative to the negative control value, SiO_2_NP treatments of 500, 1000, or 2000 mg/kg caused no significant increase in MNPCE numbers in mice.

### 3.5. SiO_2_NPs Exposure Results In Vitro Comet Assay

**Figure 3:**
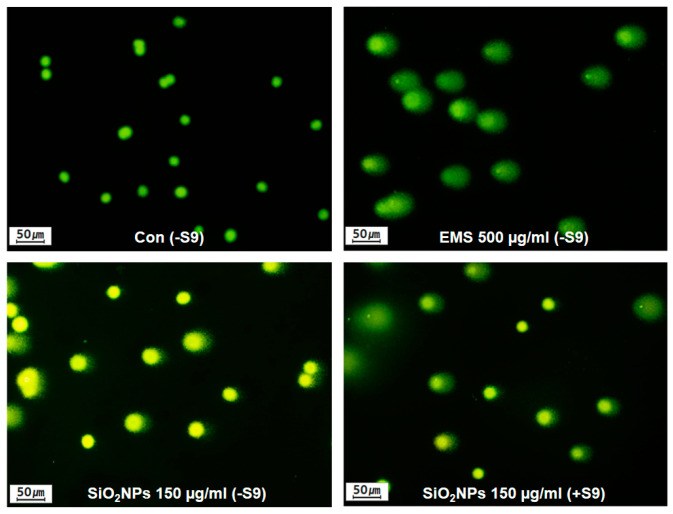
Effects of SiO_2_NPs on DNA damage in CHL cells. Representative in vitro comet assay after the treatment with 150 μg/mL SiO_2_NPs or 500 µg/mL ethyl methanesulfonate (EMS) for 24 h in the absence of S9 mix and 150 μg/mL SiO_2_NPs for 4 h in the presence of S9 mix.

Direct measurement of DNA damage was conducted using the comet assay to analyze tail parameters in CHL cells treated with SiO_2_NPs at different concentrations. The comet parameters of tail length and tail moment representing DNA migration during electrophoresis have been developed by Singh et al. [31] and Olive et al. [32] to classify undamaged (no migration) or damaged (migrated DNA) cells. The cells treated with EMS as the positive control showed a strongly positive response in the comet assay, indicating significant increases in DNA damage. Based on the results of the MTT assay in CHL cells (Table S2), the highest concentration of SiO_2_NPs selected for in vitro comet assay was 150 μg/mL showing more than 70% of cell viability. SiO_2_NPs caused significant increases in DNA in the tail and Olive tail moment at doses of 37.5, 75, and 150 μg/mL for 4 or 24 h in the absence of metabolic activation. With S9, the tail and Olive tail moment were significantly increased for 150 μg/mL SiO_2_NP treatment for 4 h (Figure 3 and Table 5).

### 3.6. SiO_2_NPs Exposure Results in GJIC Analysis

**Figure 4:**
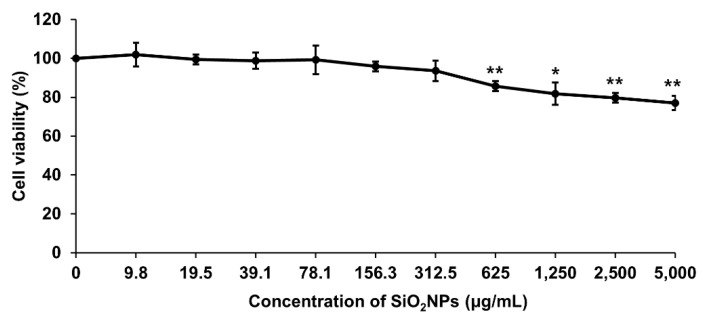
Effects of SiO_2_NPs on cell viability in WB-F344 cells. Data expressed as means ± SD (* *p* < 0.05 and ** *p* < 0.01).

As WB-F344 rat liver epithelial cells are stem-like cells and inherently express abundant Cx43 unlike other cell lines [22,40], this cell line was selected for GJIC analysis in the present study. Cell viabilities were measured using a trypan blue exclusion assay following the treatment of SiO_2_NPs in the range of 9.8–5000 µg/mL. Since the mean viability of SiO_2_NPs at a concentration of 5000 µg/mL was 76.99% of the mean value of control (Figure 4), 5000 μg/mL of SiO_2_NPs was selected as the highest concentration for the subsequent GJIC experiments. 

**Figure 5:**
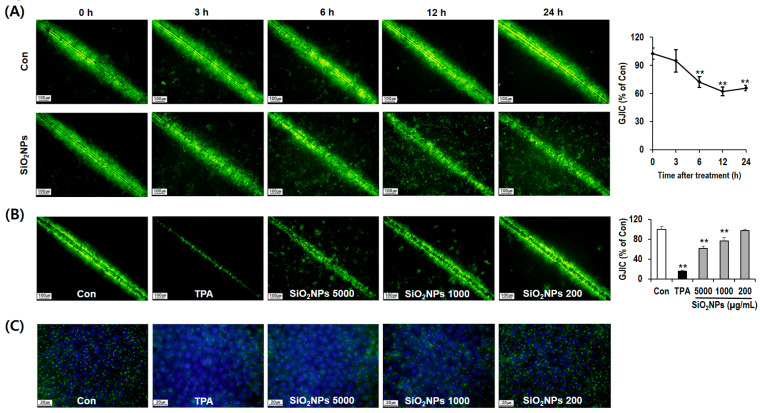
Effects of SiO_2_NPs on gap junctional intercellular communication (GJIC) and Cx43 protein in WB-F344 cells. (**A**) Representative scrape loading/dye transfer (SL/DT) assay for time-course study on GJIC and quantitative analysis after the treatment with 5000 µg/mL SiO_2_NPs. (**B**) Representative SL/DT assay for dose–response study on GJIC and quantitative analysis after the treatment with SiO_2_NPs for 12 h at doses of 200, 1000, and 5000 µg/mL. Then, 10 ng/mL 12-O-tetradecanocylphorbol-13-acetate (TPA) was treated as a positive control. (**C**) Effects of SiO_2_NPs on the distribution of Cx43. Distribution of Cx43 levels (green) was detected by immunofluorescence staining after the treatment with SiO_2_NPs for 12 h at doses of 200, 1000, and 5000 µg/mL. Then, 10 ng/mL TPA was treated as a positive control. Nuclei were stained with 4′,6-Diamidino-2-phenylindole dihydrochloride (DAPI )(blue). Bar represents 1.0 mm. Data expressed as means ± SD (* *p* < 0.05 and ** *p* < 0.01).

In a time-course study using the SL/DT method, a simple functional assay for the assessment of GJIC [34], 5000 µg/mL of SiO_2_NPs inhibited GJIC the most (by 37.75%) at 12 h compared with other time points, indicating that the optimal time point was 12 h for the dose–response study of GJIC (Figure 5A). Additionally, we observed a dose-dependent suppression of dye transfer through functional gap junctions following the SiO_2_NP treatments at 200 (3.17%), 1000 (23.35%), and 5000 µg/mL (38.57%) for 12 h (Figure 5B).

**Figure 6:**
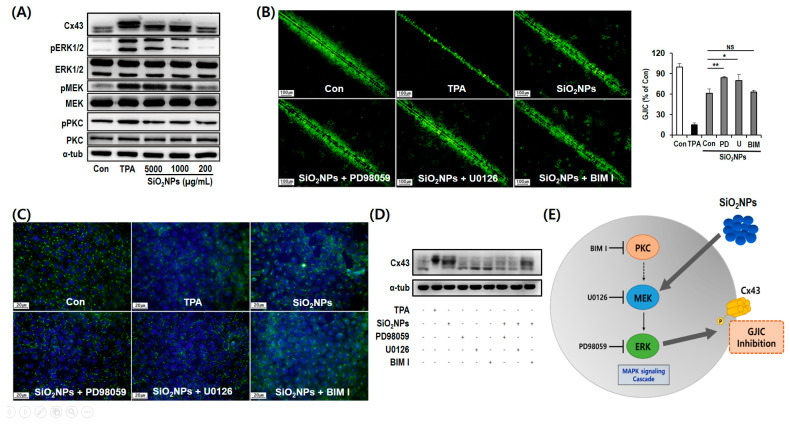
Effects of SiO_2_NPs on phosphorylation Cx43 in WB-F344 cells. (**A**) Effects of SiO_2_NPs on Cx43 phosphorylation and MAPK pathway activation in WB-F344 cells. Representative Western blot assay after the treatments with 200, 1000, or 5000 µg/mL SiO_2_NPs and 10 ng/mL TPA for 12 h. (**B**–**D**) Effects of MAPKs pathway inhibitors on SiO_2_NP-induced GJIC inhibition in WB-F344 cells. The cells were pretreated with 50 µM ERK inhibitor PD98059, 10 µM MEK inhibitor U0126, and 10 µM PKC inhibitor BIM I for 30 min before the treatment with 5000 µg/mL SiO_2_NPs for 12 h. In total, 10 ng/mL TPA was treated as a positive control. (**B**) Representative images for SL/DT assay. NS, not significant. (**C**) Representative images for immunofluorescence staining. (**D**) Representative images for Western blot assay. (**E**) Schematic representation of the inhibition of GJIC by the SiO_2_NPs. Data expressed as means ± SD (* *p* < 0.05 and ** *p* < 0.01). Bar represents 1.0 mm.

Immunofluorescence staining was performed to determine the expression and localization of Cx43 protein, one of the major gap junction proteins among connexin isotypes [41]. As a result, the expression of Cx43 decreased in a dose-dependent manner after treatment with SiO_2_NP for 12 h (Figure 5C). Especially, SiO_2_NP treatment caused reductions in Cx43 localization at regions of cell–cell contact. Since Cx43 phosphorylation is important to evaluate functional gap junctions [41], we further conducted Western blot analysis of Cx43 to elucidate the mechanism of GJIC inhibition by SiO_2_NPs. Among the three major bands (P0, P1, and P2) on the membrane at 39–44 kDa with the Cx43 antibody, the mobility shifts ranging from band P0 to P1 or P2 indicate the hyperphosphorylation of Cx43 [42]. In the present study, Cx43 was detected on the only P0 and P1 bands in untreated control cells whereas SiO_2_NP treatments dose-dependently increased the phosphorylation of Cx43 (Figure 6A).

### 3.7. Mechanism of GJIC Inhibition by SiO_2_NPs

Since the activation of the MAPK pathway is closely associated with GJIC regulation [41,43], we analyzed the phosphorylation state of ERK1/2, MEK, and PKC to identify protein kinases related to Cx43 phosphorylation. In this study, SiO_2_NPs dose-dependently activated the phosphorylation of ERK1/2 and MEK kinases, but not PKC (Figure 6A). We further confirmed the effects of ERK1/2 and MEK kinases on GJIC using the respective inhibitors. In SL/DT assay, pretreatment with ERK1/2 inhibitor (PD98059) and MEK inhibitor (U0126) significantly restored SiO_2_NP (5000 µg/mL)-induced inhibition of dye transfer (Figure 6B). Further, the SiO_2_NP-induced alterations of expression and plasma membrane localization of Cx43 were significantly blocked, at least partially, by pretreatment with PD98059 and U0126 in immunofluorescence staining whereas PKC inhibitor BIM I did not affect these SiO_2_NP-mediated Cx43 alterations (Figure 6C). SiO_2_NP-induced phosphorylation of Cx43 was also significantly attenuated by PD98059 and U0126 pretreatment before SiO_2_NPs. However, no attenuation of the Cx43 phosphorylation was detected by BIM I pretreatment (Figure 6D).

## 4. Discussion

The growth of nanotechnology in many fields has raised the risk of unexpected adverse effects associated with nanoparticle exposures. Especially, amorphous silicas have generated substantial interest in nanomedicine [14,15,17,18,44], but some amorphous samples were found to induce cellular damage [45] and proinflammatory responses [46]. Size-dependent pulmonary injury and neutrophilic infiltration were observed in mice that received silica particles via oropharyngeal aspiration [47]. The DNA-damaging potential of amorphous silica was also shown to be size-dependent [48]. Despite these growing safety concerns of SiO_2_NPs, its toxicity remains inconclusive due to the lack of comprehensive analysis according to a standardized protocol compared with crystalline silica. In particular, the nanoparticles can be accumulated in the organs by taking a long period to clear, and they may lead to genotoxicity and carcinogenicity as persistent bioaccumulative toxic substances. In fact, Guo et al. [49] reported that the chronic exposure to amorphous SiO_2_NPs caused malignant transformation of human lung epithelial cell via aberrant p53 signaling. In addition, Xie et al. [50] investigated the tumorigenic mechanisms of SiO_2_NPs associated with oxidative stress and oxidative phosphorylation. Therefore, this study aimed to elucidate the genotoxic and non-genotoxic tumor-promoting effects of SiO_2_NPs along with the possible mechanisms.

We first tested the clastogenic and mutagenic potentials of SiO_2_NPs based on three regulatory genotoxicity studies (a bacterial reverse mutation test, an in vitro chromosome aberration assay, and an in vivo micronucleus assay) according to the OECD test guidelines [27,28,29] under the principles of Good Laboratory Practices [21]. First, no significant SiO_2_NP-related increases in revertant colonies were observed in the Ames test used to detect mutagenic effects including base-pair substitutions, frameshift mutations, or oxidative and cross-linking mutations [51,52,53,54]. Second, we evaluated the clastogenic activity of SiO_2_NPs with an in vitro mammalian chromosomal aberration test to identify chromosomal breaks, fragments, and exchanges in cultured mammalian cells [55] and in vivo bone marrow micronucleus assay as an indirect indicator of quantitative chromosomal disorders [56], and found that that the SiO_2_NPs did not induce any significant changes regardless of metabolic activation. Even though the aforementioned three genotoxicity tests are the most commonly used screening methods approved by the OECD, some can be inappropriate for nanoparticles. For example, the Ames test has been predominantly negative for various kinds of nanoparticles due to limited or no diffusion through the bacterial wall [57,58,59,60]. Therefore, we additionally conducted the in vitro comet assay to quantify DNA damage expressed as % tail DNA and Olive tail moment, which has higher sensitivity for detecting low levels of DNA damage identifying at the single-cell level [32]. Noteworthy, the SiO_2_NP-induced DNA breakage was detected in this study although it was negative in an Ames test, a chromosome aberration assay, and a micronucleus assay. This result can support previous reports on in vitro comet assay involving mammalian cell lines, which has been known to be more sensitive for the genotoxicity of nanoparticles in comparison with other genotoxicity tests [32,57,59] although it is necessary to have a comprehensive evaluation using a battery of different short-term genotoxicity tests together with interpretation criteria to avoid interference and possible false-negative results.

Dysfunctional GJIC has been associated with several diseases such as neuropathy [61], hereditary deafness [62], cataract [63], skin disease [64], and heart disease [65]. Additionally, it is significantly linked to carcinogenesis because it disrupts homeostasis, which modulates cell proliferation and growth in multicellular organisms [66]. Similarly, most tumor cells exhibit dysfunctional GJIC [24] and numerous studies reported that oncogene transfection and treatment with tumor promoters, such as TPA, decreased the GJIC [22,23]. In addition to the genotoxic carcinogen, there are non-genotoxic carcinogens, which produce cancer through secondary mechanisms unrelated to direct gene damage, such as hormonal effects, cytotoxicity, or epigenetic changes [67]. In the present study, to identify the non-genotoxic tumor-promoting potential of SiO_2_NPs, we performed the SL/DT assay, which is the most frequently used assay for the assessment of GJIC [34] and found that SiO_2_NPs inhibited dye transfer at doses selected by cell viability assay. Consistently, immunofluorescence staining indicated the dose-dependent reduction in the expression and plasma membrane localization of Cx43 following SiO_2_NPs treatments, supporting our results from SL/DT assay since Cx43 internalization is linked to GJIC dysregulation via protein degradation [42].

We investigated the mechanisms underlying the altered expression of Cx43 by Western blot. First, the phosphorylation of Cx43 in the plasma membrane can lead to inhibition of GJIC through changes in the assembly, stability, and functionality of gap junctions [41]. Second, the low transcriptional output can also be linked to a decreased level of the main gap junction proteins such as Cx43 [68]. Numerous studies have shown that the phosphorylation state of connexin is affected by several exogenous chemicals such as 18α-glycyrrhetinic acid, TPA, dieldrin, and heptachlor epoxide [22,41]. In the present study, SiO_2_NPs dose-dependently inhibited GJIC along with the phosphorylation of Cx43. To investigate the mechanism of SiO_2_NP-induced Cx43 phosphorylation involved in GJIC inhibition, we identified the kinases, which were phosphorylated in the cells treated with SiO_2_NPs. Activation of the MEK/ERK/MAP kinase signal transduction pathway has been known to regulate cellular proliferation, survival, and differentiation [69]. Increased levels of MEK and ERK phosphorylation have been reported in response to treatment with well-known carcinogens such as TPA and dichloro-diphenyl-trichloroethane (DDT) [70,71]. Besides, PKC is known to activate MAPK signaling pathway via activation of MEK [72]. In this study, the inhibition of GJIC was found to be mediated by the phosphorylation of Cx43 through activation of the MAPK pathway. Moreover, the inhibitory activity of SiO_2_NPs on GJIC was restored by ERK inhibitor and MEK inhibitor, but not by PKC inhibitor, indicating a positive relationship between SiO_2_NP-induced GJIC suppression and MAPK pathway. These led us to the novel discovery on the non-genotoxic carcinogenic potential of SiO_2_NPs and its mechanism involved in abnormal GJIC function.

Many studies have investigated the influence of physicochemical properties of silica nanoparticles such as size, surface charge, geometry, and porosity on their toxicity. Ariano et al. [73] reported that smaller sized silica nanoparticles had a greater ability to induce toxicity and oxidative stress (size ranges from 20 nm to 500 nm) in human hepatoma HepG2 cells. In addition, Yu et al. [74] demonstrated that the porosity and surface charge of silica nanoparticles were found to be important features to control the plasma membrane damage and cytotoxicity in macrophages. In this study, our findings suggest that the in vitro comet assay and GJIC evaluation are appropriate methods for screening of the genotoxic and non-genotoxic carcinogenic potentials of various nanoparticles including SiO_2_NPs. Nonetheless, conclusions regarding the correlation of the physicochemical properties of the SiO_2_NPs and its side effects based on the results of comet assay and GJIC analysis would require further verification.

## 5. Conclusions

In the present study, we have herein revealed that the SiO_2_NPs induced dose-dependent DNA damage in the comet assay in mammalian cell lines despite negative responses of SiO_2_NPs detected in the Ames test, chromosomal aberration assays, or micronucleus assays. Further, SiO_2_NP-induced Cx43 phosphorylation and subsequent abnormal GJIC occurred in carcinogenesis through activation of the MAPK pathway (Figure 6E). Taken together, this study provides useful information about the potential risk of human exposure to SiO_2_NPs at different concentrations based on the results of comet assay and GJIC analysis. 

## Figures and Tables

**Table 1:** Physico-chemical characterization of SiO_2_NPs.

Particle Diameter (nm) ^a^	Hydrodynamic Size (nm) ^b^	Zeta-Potential (mV) ^c^
18.54 ± 5.05	68.78 ± 2.43	−32.07 ± 0.32

^a^ Diameter determined in deionized water by TEM**.** ^b^ Hydrodynamic size determined in deionized water by DLS**.** ^c^ Zeta-potential determined by DLS**.** Data are represented as the mean ± standard deviation of triplicate measurements.

**Table 2:** Results of *S. typhimurium* reversion assay with SiO_2_NPs.

S9	Substance	Dose (µg/100 µL/plate)	His^+^ Revertant Colony/Plate				
			TA98	TA100	WP2uvrA	TA1535	TA1537
-	Distilled water ^a^	-	28 ± 5.1	125 ± 8.7	22 ± 2.1	12 ± 1.7	16 ± 3.0
	2-nitrofluorene ^b^	10	640 ± 54.1 *	-	-	-	-
	Sodium azide ^b^	5	-	884 ± 29.8 *	-	-	-
	Mitomycin C ^b^	0.5	-	-	413 ± 12.5 *	-	-
	Sodium azide ^b^	0.5	-	-	-	419 ± 22.1 *	-
	9-aminoacridine ^b^	80	-	-	-	-	894 ± 44.5 *
	SiO_2_NPs	1250	29 ± 1.7	130 ± 2.6	26±2.6	8 ± 2.5	17 ± 1.2
		2500	30 ± 4.5	129 ± 5.0	25 ± 0.6	11 ± 4.2	18 ± 2.6
		5000	24 ± 4.0	130 ± 8.5	22 ± 2.5	9 ± 1.2	18 ± 2.5
+	Distilled water ^a^	-	31 ± 7.4	117 ± 6.6	38 ± 1.5	14 ± 2.5	20 ± 2.1
	2-aminoanthracene ^b^	2.0	405 ± 16.5 *	431 ± 14.0 *	-	-	-
		5.0	-	-	397 ± 19.9 *	411 ± 8.0 *	408 ± 16.1 *
	SiO_2_NPs	1250	34 ± 0.6	113 ± 3.1	30 ± 7.5	14 ± 0.6	18 ± 1.5
		2500	34 ± 5.9	114 ± 4.0	35 ± 1.0	12 ± 2.5	18 ± 0.6
		5000	30 ± 7.2	121 ± 11.9	31 ± 2.3	14 ± 3.1	19 ± 1.0

^a^ Negative control. ^b^ Positive control. * Significantly different from the negative control group (*p* < 0.05).

**Table 3:** Results of chromosomal aberration induced by SiO_2_NPs.

Substance	Dose (µg/mL)	Number of Cells Scored	No. of Cells with Aberrations		
			−S9		+S9
			6 h	24 h	6 h
MEM ^a^	-	200	0.5 ± 0.71	1.0 ± 0.00	0
Mitomycin C ^b^	0.1	200	26.0 ± 2.83 *	36.5 ± 2.12 *	-
Cyclophosphamide ^b^	5	200	-	-	48.5 ± 0.71 *
Distilled water	-	200	0	0.5 ± 0.71	0.5 ± 0.71
SiO_2_NPs	133.38	200	0	0.5 ± 0.71	0.5 ± 0.71
	153.39	200	1.0 ± 1.41	0.5 ± 0.71	0
	176.40	200	0	0	0

^a^ Minimum essential medium (negative control). ^b^ Positive control. * Significantly different from the negative control group (*p* < 0.05).

**Table 4:** Micronucleated polychromatic erythrocytes in mice bone marrow following treatment with SiO_2_NPs.

Substance	Dose (mg/kg BW)	Number of Mice	MNPCE ^c^	PCE/(PCE + NCE) ^d^
Distilled water ^a^	-	5	2.0 ± 1.00	49.4 ± 4.40
SiO_2_NPs	500	5	2.0 ± 1.22	42.9 ± 10.99
	1000	5	5.4 ± 2.19	46.2 ± 4.25
	2000	5	3.8 ± 0.84	45.7 ± 4.53
Mitomycin C ^b^	2	5	137.0 ± 11.96 **	39.8 ± 2.97 *

^a^ Negative control. ^b^ Positive control. ^c^ Polychromatic erythrocyte with micronuclei was calculated from 2000 polychromatic erythrocytes. ^d^ The ratio of polychromatic erythrocytes to all erythrocytes (polychromatic + normochromatic) (%). * Significantly different from the negative control group (*p* < 0.05). ** Significantly different from the negative control group (*p* < 0.01). MNPCE: micronucleated polychromatic erythrocytes, PCE: polychromatic erythrocytes, NCE: normochromatic erythrocytes

**Table 5:** Results of in vitro comet assay with SiO_2_NPs.

Substance	Dose (µg/mL)	S-9 Mix	Time (h)	% Tail DNA	Olive Tail Moment	Relative Cell Count (%)
Distilled water ^a^	-	-	4	5.66 ± 3.66	0.46 ± 0.31	100
SiO_2_NPs	37.5		4	12.16 ± 6.02 **	1.29 ± 1.05 **	98
	75.0		4	13.94 ± 6.66 **	1.40 ± 1.13 **	83
	150.0		4	13.11 ± 6.12 **	1.19 ± 1.09 **	78
EMS ^b^	500		4	25.18 ± 12.13 **	2.86 ± 2.34 **	73
Distilled water ^a^	-	-	24	5.74 ± 3.32	0.48 ± 0.45	100
SiO_2_NPs	37.5		24	11.65 ± 6.39 **	1.11 ± 1.02	95
	75.0		24	12.17 ± 6.64 **	1.07 ± 1.01	86
	150.0		24	20.51 ± 10.47 **	2.50 ± 1.98 **	77
EMS ^b^	500		24	39.44 ± 14.26 **	8.29 ± 6.25 **	67
Distilled water ^a^	-	+	4	5.20 ± 3.20	0.43 ± 0.32	100
SiO_2_NPs	37.5		4	6.13 ± 3.34	0.47 ± 0.38	94
	75.0		4	7.47 ± 3.8	0.62 ± 0.53	85
	150.0		4	10.10 ± 5.32 **	1.00 ± 0.84 *	79
EMS ^b^	500		4	24.59 ± 11.21 **	3.12 ± 2.34 **	76

^a^ Negative control. ^b^ Positive control. * Significantly different from the negative control group (*p* < 0.05). ** Significantly different from the negative control group (*p* < 0.01).

## Supplementary Materials

The following are available online at https://www.mdpi.com/1999-4923/12/9/826/s1, Table S1. Results of cytotoxicity test treated with SiO_2_NPs, Table S2. Results of MTT assay in CHL cells treated with SiO_2_NPs, Table S3. Effects of SiO_2_NPs on the body weight changes in micronucleus assay.
