# Supplementary material for: Toxicologic Evaluation for Amorphous Silica Nanoparticles: Genotoxic and Non-Genotoxic Tumor-Promoting Potential
Source: Pharmaceutics. 2020 Aug 29;12(9):826. doi: 10.3390/pharmaceutics12090826 (PMC7559769; doi:10.3390/pharmaceutics12090826)
Supplement: Supplementary file 1 [file pharmaceutics-12-00826-s001.pdf]

pharmaceutics

Supplementary Materials: Toxicologic Evaluation for Amorphous Silica Nanoparticles: Genotoxic and Non-Genotoxic Tumor-Promoting Potential

Gwang-Hoon Lee, Yun-Soon Kim, Euna Kwon, So-Hee Kim, Jun-Won Yun and Byeong-Cheol Kang

Table S1. Results of cytotoxicity test treated with SiNPs.

S9	Substance	Dose (μg/plate)	His+ Revertant Colony/Plate (TA100)
-	Distilled water[a]	-	123 ± 5.1
	SiNPs	1250	123 ± 3.1
		2500	121 ± 2.1
		5000	120 ± 2.5
+	Distilled water[a]	-	145 ± 2.1
	SiNPs	1250	132 ± 1.5
		2500	136 ± 3.0
		5000	132 ± 3.0

Table S2. Results of MTT assay in CHL cells treated with SiNPs.

Substance	Dose (μg/mL)	Absorbance	Cell Survival Rate (%)
Distilled water[a]	0.0	0.533 ± 0.015	100.00
SiNPs	74.81	0.581 ± 0.017	109.17
	82.29	0.597 ± 0.053	112.18
	90.52	0.609 ± 0.021	114.37
	99.57	0.588 ± 0.015	110.36
	109.53	0.572 ± 0.016	107.48
	120.48	0.529 ± 0.008	99.28
	132.53	0.543 ± 0.008	102.03
	147.78	0.489 ± 0.006	91.89
Distilled water[a]	0.0	0.543 ± 0.016	100.00
SiNPs	160.36	0.445 ± 0.006	81.99
	176.40	0.398 ± 0.009	73.21
	194.04	0.256 ± 0.008	47.13
	213.44	0.212 ± 0.014	39.09
	234.79	0.139 ± 0.005	25.59
	258.26	0.097 ± 0.010	17.80
	284.09	0.071 ± 0.015	13.07
	312.50	0.076 ± 0.019	13.93

[a] Negative control

Table S3. Effects of SiNPs on the body weight changes in micronucleus assay

Substance	Dosage (mg/kg BW)	Number of Mice	Body Weight (g)				
			0 day	1 day	2 day	3 day	Sacrifice
Distilled water[a]	0.0	5	35.73 ± 1.572	36.16 ± 2.013	36.56 ± 2.003	36.25 ± 1.824	37.86 ± 2.135
	500	5	35.26 ± 1.268	35.92 ± 1.570	36.07 ± 1.628	35.82 ± 1.595	36.78 ± 1.887
SiNPs	1000	5	35.23 ± 1.613	35.68 ± 2.114	35.99 ± 2.092	35.78 ± 2.288	37.00 ± 2.321
	2000	5	35.40 ± 1.695	35.67 ± 1.614	36.31 ± 1.623	36.11 ± 1.749	37.18 ± 2.159
Mitomycin C [b]	2	5	35.36 ± 1.724	35.95 ± 1.663	36.41 ± 1.710	36.07 ± 1.677	37.45 ± 1.908

[a] Negative control, [b] Positive control
